# WO_3_ Nanorods Decorated with Very Small Amount of Pt for Effective Hydrogen Evolution Reaction

**DOI:** 10.3390/nano13061071

**Published:** 2023-03-16

**Authors:** Giacometta Mineo, Luca Bruno, Elena Bruno, Salvo Mirabella

**Affiliations:** 1 Department of Physics and Astronomy “Ettore Majorana”, University of Catania, Via S. Sofia 64, 95123 Catania, Italy; giacometta.mineo@dfa.unict.it (G.M.); luca.bruno@dfa.unict.it (L.B.); elena.bruno@dfa.unict.it (E.B.); 2 IMM-CNR, Via S. Sofia 64, 95123 Catania, Italy

**Keywords:** hydrogen evolution reaction, ultra-low Pt amount, WO_3_, nanorods, electrochemistry

## Abstract

The electrochemical hydrogen evolution reaction (HER) is one of the most promising green methods for the efficient production of renewable and sustainable H_2_, for which platinum possesses the highest catalytic activity. Cost-effective alternatives can be obtained by reducing the Pt amount and still preserving its activity. The Pt nanoparticle decoration of suitable current collectors can be effectively realized by using transition metal oxide (TMO) nanostructures. Among them, WO_3_ nanorods are the most eligible option, thanks to their high stability in acidic environments, and large availability. Herein, a simple and affordable hydrothermal route is used for the synthesis of hexagonal WO_3_ nanorods (average length and diameter of 400 and 50 nm, respectively), whose crystal structure is modified after annealing at 400 °C for 60 min, to obtain a mixed hexagonal/monoclinic crystal structure. These nanostructures were investigated as support for the ultra-low-Pt nanoparticles (0.2–1.13 μg/cm^2^): decoration occurs by drop casting some drops of a Pt nanoparticle aqueous solution and the electrodes were tested for the HER in acidic environment. Pt-decorated WO_3_ nanorods were characterized by performing scanning electron microscopy (SEM), X-ray diffraction analysis (XRD), Rutherford backscattering spectrometry (RBS), linear sweep voltammetry (LSV), electrochemical impedance spectroscopy (EIS) and chronopotentiometry. HER catalytic activity is studied as a function of the total Pt nanoparticle loading, thus obtaining an outstanding overpotential of 32 mV at 10 mA/cm^2^, a Tafel slope of 31 mV/dec, a turn-over frequency of 5 Hz at −15 mV, and a mass activity of 9 A/mg at 10 mA/cm^2^ for the sample decorated with the highest Pt amount (1.13 μg/cm^2^). These data show that WO_3_ nanorods act as excellent supports for the development of an ultra-low-Pt-amount-based cathode for efficient and low-cost electrochemical HER.

## 1. Introduction

The increasing global energy demand, the finite supply of fossil fuels, and the related global warming and environmental pollution pushes the attention of the scientific community to the development of renewable and sustainable energy sources. In this scenario, H_2_ is a key player since it is characterized by high reactivity and its energy content is three times higher than gasoline [1]. The H_2_-based economy represents a valid green alternative to solve the problems related to fossil fuel combustion. H_2_ naturally exists combined with other elements and not in the pure elemental gas form [2], so the production of H_2_ gas, starting from H-based compounds, is fundamental for the development of an efficient H_2_-based economy.

One of the most sustainable and eco-friendly methods to produce green H_2_ is electrochemical water splitting, which coincides with the separation of water in H_2_ and O_2_ (hydrogen and oxygen evolution reaction, respectively), thanks to an external potential bias [3,4,5]. Platinum is the most active catalyst for the hydrogen evolution reaction (HER) process, especially in acidic environments [6]. Nevertheless, the limited worldwide supply and the high cost of Pt hinders its use for large-scale H_2_ production through electrochemical water splitting [7,8]. For the establishment of a carbon-neutral economy, a significant reduction of the Pt load for HER is urgently needed [9]. A potential solution is the development of a low-cost and efficient support for Pt to reduce its utilization. Transition metal oxide (TMO)-based nanostructures possess high electrochemical stability in both acidic and alkaline media, are earth abundant, and their electrochemical properties depend on morphology and crystal structure. They can be useful as Pt supports, giving a high surface to volume ratio if the charge exchange towards the catalytic noble element is not hindered. Decoration with very low amounts of Pt nanoparticles allows the exploitation of the synergistic effect between Pt nanoparticles and TMO-based nanostructures. This strategy enables the lowering of the total production cost, without affecting the Pt electrocatalytic efficiency. Xie et al. [10] synthesized MoO_2_ on multiwalled carbon nanotubes (MWCNTs) and decorated these nanocomposites with a low amount of Pt nanoparticles (0.47 mg/cm^2^). The electrochemical analysis in 0.5 M H_2_SO_4_ confirms the excellent activity of the decorated nanocomposite, which shows an overpotential at 10 mA/cm^2^ of 60 mV, compared to those of the bare composite which is about 500 mV.

Several earth-abundant TMO-based electrocatalysts have been studied for the activation of efficient HER in acidic conditions [4,6,11,12]. Among them, WO_3_ represents a suitable candidate thanks to its large availability and high electrochemical stability at low pH, especially in the nanostructured form (nanorods, nanowires, nanosheets) [13,14,15]. Unfortunately, in their pristine form, WO_3_ nanostructures possesses a poor electron transport ability and few active sites for hydrogen ion absorption, which result in a poor HER ability [7,16,17]. Many efforts have been made with the aim of improving the HER catalytic activity of WO_3_ nanostructures, such as the realization of heterostructures by coupling WO_3_ with other transition-metals-based materials (WS_2_, WSe_2_, or WC) [18,19,20] and embedding with carbon-based materials [21,22]. In our previous work [13], we proposed a low cost and simple approach to improve the HER catalytic activity of WO_3_, reducing the overpotential at 10 mA/cm^2^ from 460 mV (for as-prepared fully hexagonal WO_3_) to 170 mV (for hexagonal/monoclinic mixed-phase WO_3_ nanorods).

Here, we report a decoration of phase engineered WO_3_ nanorods with an ultra-low amount of Pt nanoparticles as a new strategy to further improve H_2_ production. The HER catalytic activity is studied as a function of Pt-loading reaching a notable overpotential of 32 mV at 10 mA/cm^2^, a Tafel slope of 31 mV/dec, and an unprecedented turn-over frequency and mass activity of 5 Hz at −15 mV and 9 A/mg at 10 mA/cm^2^, respectively.

## 2. Materials and Methods

### 2.1. Synthesis of WO_3_ Nanorods and Pt Nanoparticles

All the reagents were purchased from Sigma-Aldrich, (St. Louis, MO, USA) and used without further purification. WO_3_ nanorods were hydrothermally synthesized according to our previous work [14,23]. In brief, the precursor solution was prepared using sodium tungstate (Na_2_WO_4_, 0.825 g) dissolved in deionized water (19 mL). Then, 3 M hydrochloric acid (HCl) and sodium chloride (NaCl) were used to acidify the solution (until a 2.2 pH) and as capping agent, respectively. The thermal treatment was conducted in a muffle by using a 25 mL autoclave, at 180 °C for 3 h. The obtained nanostructures were collected and washed by centrifugating with water and ethanol several times (6000 RPM for 10 min). The powders were calcinated on a hot plate in air at 400 °C, as described in our previous work [13], to obtain a partial hexagonal to monoclinic phase transition until reaching similar contents. The annealing time was set as 60 min, so that the hexagonal and monoclinic crystallites phases had comparable volumes [14].

Pt nanoparticles were synthesized according to Bruno et al. [24], thus using a room temperature and green chemical reduction method. Ascorbic acid (AA, 30 μL of a 33 mM solution) was used as reducing acid and was dispersed in a H_2_PtCl_6_ solution (30 mL of 0.2 mM solution). The obtained dispersion was stirred for 5 min.

The Pt electrode was prepared by sputtering deposition on a graphene paper ((GP, 2 × 1 cm^2^, 240 μm thick, Sigma Aldrich, St. Louis, MO, USA)) substrate, using a sputter apparatus Emitech K550X A (Ashford Kent, UK), in which the GP was the cathode, facing the Pt source (purity of 99.999%). Next, 100 nm of Pt film was deposited on the GP substrate, with a covered area of 1 cm^2^, by setting the emission current at 50 mA and the deposition time at 16 min.

### 2.2. Electrode Preparation

WO_3_ nanorods powder was dissolved in water (4 mg/mL) and sonicated for 10 min to reach a homogeneous aqueous dispersion. GP was used as substrate after a polishing procedure with water and ethanol. The WO_3_-based dispersion was used for drop coating the GP substrate, thus covering a 0.3 cm^2^ area (drying in air for 20 min at 70 °C allowed solvent evaporation). A Mettler Toledo MX5 Microbalance (sensitivity: 0.01 mg) was used to measure the mass of the electrode (substrate + WO_3_ nanorods) and of the bare substrate. Some drops of the Pt dispersion (5 μL volume) were drop coated on the WO_3_ nanorod-based electrodes, which were labeled 5Pt-WO_3_, 10Pt-WO_3_, and 20Pt-WO_3_, respectively, depending on the used drops number.

### 2.3. Characterization of the Pt-Decorated WO_3_ Nanorods

Film structure was analyzed through X-ray Diffraction (XRD) by using a Smartlab Rigaku diffractometer (Rigaku Corporation, Tokyo, Japan), at a grazing incidence of 0.5°, equipped with a rotating anode of Cu Kα radiation operating at 45 kV and 200 mA. The scans were acquired from 10° to 70° with a step of 0.02°. The morphological analyses were carried out using a Gemini Field Emission SEM Carl Zeiss SUPRATM 25 (FEG-SEM, Carl Zeiss Microscopy GmbH, Jena, Germany) scanning electron microscope (SEM) in IN-LENS mode. For the determination of Pt content, Rutherford backscattering spectrometry (RBS, 2.0 MeV He + beam at normal incidence) with a 165° backscattering angle was employed, using a 3.5 MV HVEE Singletron accelerator. RBS spectra were analyzed using XRump software (version 0.91) [25]. The electrochemical measurements were performed at room temperature using a potentiostat (VersaSTAT 4, Princeton Applied Research, Oak Ridge, TN, USA) and a three-electrode setup with a graphite rod electrode as a counter electrode (to avoid Pt contamination), a saturated calomel electrode (SCE) as reference, and the Pt-decorated WO_3_ electrodes as working electrodes, in a 1 M H_2_SO_4_ supporting electrolyte.

### 2.4. Electrochemical Measurements

Electric current values were normalized to the geometrical-immersed surface area of each electrode. The conversion of the measured potential vs. *SCE* into the reversible hydrogen electrode (*RHE*) was carried out according to the Nernst equation [19,26]:

(1) $$E_{RHE}^{\prime }=E_{SCE}^{\Theta }+E_{SCE}\times 0.059\times pH$$

where $E_{SCE}^{\Theta }$ is the standard potential of the *SCE* electrode at 25 °C (0.241 V) and $E_{SCE}$ is the measured potential vs. *SCE*. The HER activities of WO_3_ and of Pt-decorated WO_3_-based electrodes were investigated using a linear sweep voltammetry (LSV) recorded at 5 mV/s from −0.2 V to −0.8 V vs. *SCE*. All the obtained potentials vs. *RHE* were manually corrected using *iR_u_* compensation as follows:

(2) $$E_{RHE}=E_{RHE}^{\prime }-iR_{u}$$

where *i* is the electrode current and *R_u_* [Ohms] is the uncompensated resistance, measured using electrochemical impedance spectroscopy (EIS) [27], which is performed from 10^5^ to 10^−1^ Hz in a potentiostatic mode with an AC voltage of 5 mV at the open circuit potential (OCP vs. *SCE*) as shown in Figure S1. The Tafel slope was defined as the slope of the linear fit of the potential vs. log(j(mA/cm^2^)) plot, according with the follow equation [28]:

(3) $$E_{RHE}=b\times \log\left(J\right)+a$$

where *E_RHE_* is the *iR_u_*-free potential (V), *b* is the Tafel slope (V/dec), *J* is the current density (mA/cm^2^), and *a* is a constant. The appropriate potential region for the Tafel slope analysis was chosen to ensure that the measured current density resulted only from the faradaic reaction which occurs during the HER mechanism [28].

## 3. Results and Discussion

### 3.1. Morphological and Structural Analysis

WO_3_ nanorods were synthesized using the hydrothermal route, and the post-synthesis thermal annealing (400 °C for 60 min) was conducted with the aim of improving the HER catalytic activity according to our previous work [14]. A partial hexagonal to monoclinic phase transition was achieved after the thermal annealing, as shown in the XRD pattern of Figure S2, in which the XRD pattern of the WO_3_ nanorods is compared with the characteristic XRD patterns of the hexagonal and monoclinic WO_3_. Our previous analysis confirmed the formation of stable hexagonal/monoclinic phase junctions in these conditions, which were able to confer a higher HER catalytic activity than that of the pure hexagonal WO_3_ nanorods. The reference intensity ratio (RIR) method was used for the calculation of phase composition in a hybrid phase structure, thus obtaining the monoclinic (hexagonal) weight ratio in the WO_3_-based nanorods of 56% (44%) (details in the Supplementary Materials).

**Figure 1:**
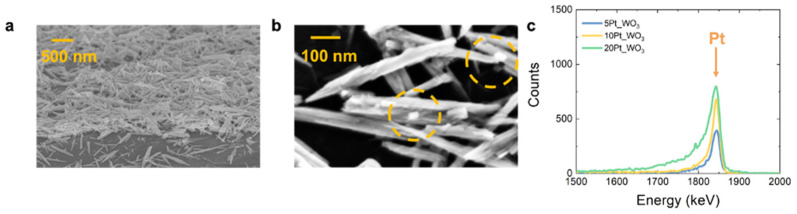
(**a**) Tilted low-magnification SEM images of bare WO_3_ electrode, composed of 3D agglomeration of nanorods; (**b**) low-magnification SEM images of WO_3_ nanorods decorated with Pt nanoparticles (highlighted in orange circles); (**c**) RBS spectrum of Pt nanoparticles on a flat Si substrate for the 5Pt_WO_3_, the 10Pt_WO_3_, and the 20Pt_WO_3_ electrodes (blue, yellow, and green lines, respectively).

Figure 1a shows a low-magnification SEM image (in tilt view) of the bare WO_3_ nanorods electrode, which is composed of randomly aligned nanorods covering the GP substrate. The image shows the role of WO_3_ nanorods in realizing a highly exposed active surface due to 3D nanorods agglomeration. Figure 1b shows low-magnification SEM images of the 10Pt-WO_3_ electrode, in which Pt nanoparticles (indicated by yellow circles, average diameter of 50 nm) are in contact with the WO_3_ nanorods. Further details of Pt nanoparticles realized with this method are reported in Refs. [24,29]. Figure S3 shows a low-magnification SEM image of the 10Pt_WO_3_ electrode in which Pt nanoparticles (highlighted in yellow) are homogeneously scattered all over WO_3_ nanorods.

By considering the assumption for which the total Pt content does not depend on the decorated substrate, the same Pt dispersion volume used for the Pt-decorated electrodes was drop casted on Si substrates and measured using RBS analysis. Figure 1c shows the enlargement of the RBS spectrum in correspondence with the Pt peak (at 1837 keV) for the 5Pt_WO_3_, the 10Pt_WO_3_, and the 20Pt_WO_3_ electrodes (blue, yellow, and green lines, respectively). The area of the peak is strictly correlated to the total Pt dose which results in 6.08 × 10^14^, 1.31 × 10^15^, and 3.51 × 10^15^ at/cm^2^ for the 5Pt_WO_3_, the 10Pt_WO_3,_ and the 20Pt_WO_3_ electrodes, respectively. Thus, considering the Pt-covered area, the total Pt mass was calculated as follows:

(4) $${Pt}_{mass}=\frac{{Dose}_{Pt}[at/{cm}^{2}]\times S\left[{cm}^{2}\right]\times M_{m(Pt)}\left[g/mol\right]}{N_{A}\left[\frac{at}{mol}\right]}$$

where *S* is the geometrical deposited area of the electrode substrate, *N_A_* is the Avogadro number and $M_{m(Pt)}$ is the *Pt* molar mass. The total mass results were 0.2, 0.43 and 1.13 μg/cm^2^ for the 5Pt_WO_3_, the 10Pt_WO_3_, and the 20Pt_WO_3_ electrodes, respectively. All these results are reported in Table 1.

### 3.2. Electrochemical Analysis

**Figure 2:**
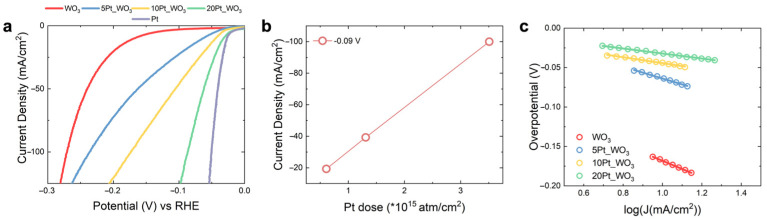
(**a**) LSV curves of the WO_3_, the 5Pt_WO_3_, the 10Pt_WO_3_, and the 20Pt_WO_3_ electrodes (red, blue, yellow, and green lines, respectively); (**b**) current density at −0.09 V as a function of Pt dose of the 5Pt_WO_3_, the 10Pt_WO_3_, and the 20Pt_WO_3_ electrodes; (**c**) Tafel plot and liner fit of WO_3_, 5Pt_WO_3_, the 10Pt_WO_3_, and the 20Pt_WO_3_ electrodes (red, blue, yellow, and green circles and lines, respectively).

Figure 2a shows the *iR_u_*-corrected LSV curve obtained for the WO_3_, the 5Pt_WO_3_, the 10Pt_WO_3_, the 20Pt_WO_3_, and the Pt electrodes (red, blue, yellow, green, purple lines) at 5 mV/s. The polarization curves of the WO_3_ differ from the other curves for the presence of a current plateau at low potentials (red curve), which results in a much lower HER.

Catalytic activity of the bare WO_3_ electrode with respect to those of the Pt-decorated electrodes. The Pt-decorated WO_3_ polarization curves show a rapid increase in the absolute value of the current density, thus suggesting a huge H_2_ production activity, which is more pronounced for the electrode with the highest Pt content (20Pt_WO_3_), as expected. The overpotential at 10 mA/cm^2^ (η) is the most common parameter used for the comparison of the HER catalytic activity. To achieve a current density of 10 mA/cm^2^, the η values are 173 mV, 63 mV, 44 mV, 32 mV, and 27 mV for the WO_3_, the 5Pt_WO_3_, the 10Pt_WO_3_, the 20Pt_WO_3_, and the Pt electrodes, respectively. This is a clear indication that decoration with an additional sub microgram amount of Pt nanoparticles per cm^2^ effectively improves the catalytic performances in HER. To highlight the role of Pt loading on HER catalytic activity, the current density at potentials above the η (–90 mV) was reported as a function of Pt dose. Figure 2b shows the result of this exercise, in which a linear dependence between the measured current density and the total Pt loading is clear. We can conclude that Pt decoration linearly increases the catalysis towards HER, showing that WO_3_ nanorods act as ideal support for Pt.

To further elaborate the difference between HER mechanism of WO_3_ and of Pt-decorated WO_3_ electrodes, Tafel analysis was carried out. Figure 2c shows the Tafel plot of the WO_3_, the 5Pt_WO_3_, the 10Pt_WO_3_, and the 20Pt_WO_3_ electrodes (red, blue, yellow, and green circles, respectively), calculated from the polarization curves reported in Figure 2a, according to Equation (3), in the faradaic potential range close to the η value of each tested electrode. The Tafel slope is defined from the linear fit of the Tafel plot (red, blue, yellow, and green lines, respectively), and results of 104 mV/dec, 75 mV/dec, 37 mV/dec and 31 mV/dec were obtained for the four samples, respectively (Table 1), thus confirming that Pt decoration results in convenient HER kinetics, since the Tafel slopes of Pt-decorated electrodes reach the theoretical Pt Tafel slope (30 mV/dec) in the same potential interval (from 0 V to −100 mV vs. RHE) [8].

**Figure 3:**
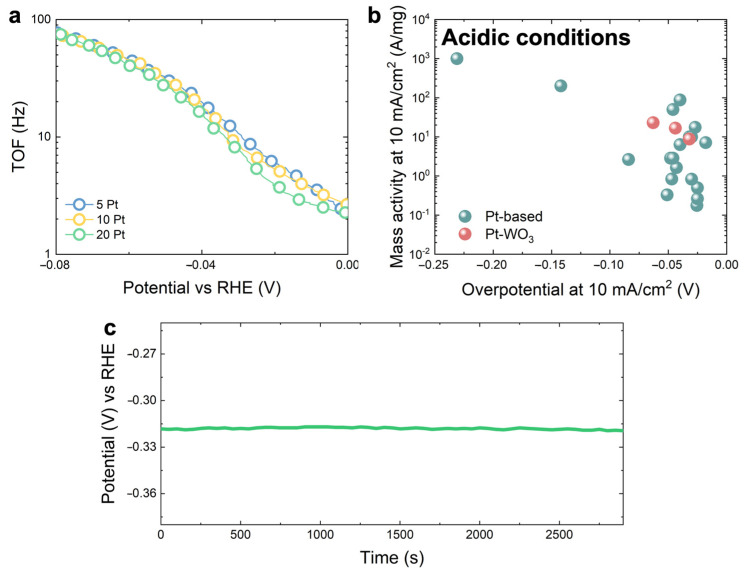
(**a**) TOF of the 5Pt_WO_3_, the 10Pt_WO_3_, and the 20Pt_WO_3_ electrodes (red, blue, yellow, and green circles, respectively) as a function of the overpotential; (**b**) comparison between the mass activity of the Pt-based electrodes reported in the literature (blue balls) [6] and our Pt-decorated WO_3_ electrodes (red balls); (**c**) chronopotentiometry analysis of the 20Pt_WO_3_ electrode.

The excellent intrinsic HER catalytic activity of the Pt-decorated WO_3_ electrodes may be further confirmed by analyzing the turn-over frequency (*TOF*) parameter, calculated starting from the polarization curve of the 5Pt_WO_3_, 10Pt_WO_3_, and 20Pt_WO_3_ electrodes (Figure 2a). The *TOF* is a marker of the intrinsic activity of the HER process, and it measures the amount of product formed or reactant consumed for a given amount of active catalyst per unit time, thus being a solid measure of the concentration of active sites [30]:

(5) $$TOF=\frac{j[A]}{xn\left[mol\right]F[C/mol]}$$

where *j* is the measure current, *x* is equal to two (number of transferred electrons for each H_2_ molecule produced), *n* is the Pt moles number, calculated from the RBS Pt dose, and *F* is the Faraday constant. Figure 3a shows the *TOF* extracted in a potential range close to the η value for the 5Pt_WO_3_, the 10Pt_WO_3_, and the 20Pt_WO_3_ electrodes (blue, yellow, and green circles). *TOF* values increase with the applied potential, as expected, and they still show comparable values between the three samples. At an overpotential lower than 0.4 V, the 20Pt_WO_3_ electrodes show a slightly lower *TOF* than that of the 5 PT_WO_3_ and of 10Pt_WO_3_ electrodes, due to its high mass (three and six times higher than the 10Pt_WO_3_ and the 5Pt_WO_3_ ones). Such a result tells us that WO_3_ nanorods act as an effective support for Pt as a HER catalyst and, more than that, it shows that even after increasing the Pt loading, a saturation effect is still not visible. A further increase in the Pt loading can be sustained by this WO_3_ nanorod support, which would give an additional increase in the current. The *TOF* value obtained at −15 mV are 5, 4, and 3 Hz for the 5Pt_WO_3_, 10Pt_WO_3_, and 20Pt_WO_3_ electrodes, respectively. These *TOF* values are comparable with those reported for commercial Pt [8], and higher if compared with the literature results on Pt-decorated nanostructures (Table 2). As a matter of fact, the catalytic activity of the Pt-decorated WO_3_-nanorod-based electrodes depends on the high concentration of exposed active absorption sites due to Pt decoration, thus highlighting the great potential of the Pt scalability process.

Mass activity is a powerful parameter used for the comparison of the HER catalytic activity of different electrodes, which allowed us to define the activity per unit mass of electrochemical active material and takes into consideration the scalability properties. Mass activity is defined as follows, thus considering the geometrical area [6]:

(6) $$Mass\;activity=\frac{j(A/{cm}^{2})}{catalist\;loading(mg/{cm}^{2})}$$

Figure 3b reports the comparison between the mass activity of the state of the art Pt-based electrodes (blue balls) and of our Pt-decorated WO_3_ electrodes (red ball) in acidic conditions, as a function of η [6]. The highest mass activity was obtained for the sample with the lowest Pt amount (5Pt_WO_3_), as expected. However, our Pt-decorated WO_3_ electrodes show at very low overpotential mass activity comparable with that of the state of the art on Pt-decorated electrodes, thus suggesting that despite the low Pt loading, the catalytic activity of our Pt-decorated WO_3_ electrodes towards HER is very promising from the perspective of the development of efficient and a low-cost HER cathode.

Stability is an important parameter which describes the quality and the activity of an electrode, especially at those potentials at which the HER mechanism occurs. Figure 3c shows the chronopotentiometry applied to study the stability of the 20Pt-WO_3_ electrode at a current density of 10 mA/cm^2^. The potential remains constant at the η value, even after 45 min of stress, thus revealing a good stability of the 20Pt_WO_3_ electrode after the activation of the HER.

Table 2 compare our results with that reported in the literature on Pt-decorated metal oxide frameworks, for HER in acidic conditions. Our electrodes show a Pt content (of the order of μg/cm^2^) 2–3 orders of magnitude lower than others, and, despite this, their electrochemical performances are much better in terms of overpotential, Tafel, TOF, and mass activity. The 20Pt_WO_3_ electrode possesses the lowest η, thus revealing a very efficient HER process, confirmed also by the high TOF value at −15 mV, which is due to the high activity of the electrochemical active sites.

## 4. Conclusions

We demonstrated the excellent HER activity of Pt-decorated phase-structure-engineered WO_3_ nanorods, creating very promising electrodes for hydrogen generation via water electrolysis. WO_3_ nanorods were synthesized using a simple hydrothermal method, followed by calcination at 400 °C, leading to a particular hexagonal/monoclinic crystal structure. The WO_3_ nanorods are active as catalysts for HER, and, still, the decoration with an ultra-low amount of Pt (about 1 μg/cm^2^)) considerably boosted the HER performance. The WO_3_ electrode decorated with 1.13 μg/cm^2^ exhibits an overpotential at 10 mA/cm^2^ of 32 mV, thanks to the synergistic effect between Pt nanoparticles and WO_3_ nanorods, as the Tafel analysis suggests. Moreover, the high TOF value towards with the mass activity of our ultra-low-amount Pt-decorated WO_3_-nanorod-based electrodes makes them potential candidates for sustainable hydrogen production, especially in the context in which the Pt use has to be controlled and reduced.

## Figures and Tables

**Table 1:** Pt dose obtained from RBS analysis and Pt loading for the Pt-decorated WO_3_-based electrodes, η, and Tafel slope of the WO_3_, the 5Pt_WO_3_, the 10Pt_WO_3,_ and the 20Pt_WO_3_ electrodes.

	Pt Dose (×10^15^ at/cm^2^)	Pt Loading (μg/cm^2^)	η (mV)	Tafel Slope (mV/dec)
WO_3_	-	-	173	104
5Pt_WO_3_	0.61	0.2	63	75
10Pt_WO_3_	1.31	0.43	44	37
20Pt_WO_3_	3.51	1.13	32	31

**Table 2:** Comparison between the state of the art on Pt-decorated metal oxide frameworks and our Pt-decorated electrodes.

	Pt Content	Electrolyte	η (mV)	Tafel Slope (mV/dec)	TOF @ − 15 mV (Hz)	Mass Activity @10 mA/cm^2^ (A/mg)	Ref.
Pt/MoO_2_/MWCNTs	0.47 mg/cm^2^	0.5 M H_2_SO_4_	60	43	2.8 @ − 50 mV	0.2	[10]
PtCoNi FNs	0.85 mg/cm^2^	0.5 M H_2_SO_4_	41	37	-	0.01	[31]
PtCoFe@CN	0.013 mg/cm^2^	0.5 M H_2_SO_4_	45	32	-	0.8	[32]
PtMoS_2_	0.036 mg/cm^2^	0.5 M H_2_SO_4_	60	96	-	0.3	[33]
PtCu nanospheres on WO_3_ nano-array	0.25 mg/cm^2^	0.5 M H_2_SO_4_	40	46	11 @ − 100 mV	2	[34]
Pt SA/m-WO_3 − x_	0.86 μg/cm^2^	0.5 M H_2_SO_4_	47	45	-	12.8 (@ 0.05 V)	[35]
5Pt_WO_3_	0.2 μg/cm^2^	1 M H_2_SO_4_	63	75	3 @ − 15 mV	23	Our work
10Pt_WO_3_	0.43 μg/cm^2^	1 M H_2_SO_4_	44	37	4 @ − 15 mV	17	Our work
20Pt_WO_3_	1.13 μg/cm^2^	1 M H_2_SO_4_	32	31	5 @ − 15 mV	9	Our work

## Data Availability

The data presented in this study are available on request from the corresponding author.

## Supplementary Materials

The following supporting information can be downloaded at: https://www.mdpi.com/article/10.3390/nano13061071/s1: Figure S1: Nyquist plot obtained from EIS analysis at open circuit potential of the WO_3_, 5Pt_WO_3_, the 10Pt_WO_3_, and the 20Pt_WO_3_ electrodes (red, blue, yellow, and green circles, respectively). Inset: Magnification of the high frequency region; Figure S2: XRD pattern of the WO_3_ nanorods (red line) compared with the characteristic patterns of hexagonal and monoclinic WO_3_: Both hexagonal (2 Ɵ = 14°, 24.37°, 26.85°, 28.22°, 33.61°, 36.57°, and 49.95°) and monoclinic (2 Ɵ = 23°, 23.50°, 24.28°, 33.12°, 33.54°, 33.83°, 34.04°, 49.74°, 55.71°) characteristic peaks appear, thus confirming the formation of stable phase junctions. Figure S3: Low magnification SEM image of the 10Pt_WO3 electrode in which the most visible Pt nanoparticles are highlighted in yellow; Description of the RIR method. References [14,36,37] are cited in the Supplementary Materials.
