# Supplementary material for: WO3 Nanorods Decorated with Very Small Amount of Pt for Effective Hydrogen Evolution Reaction
Source: Nanomaterials (Basel). 2023 Mar 16;13(6):1071. doi: 10.3390/nano13061071 (PMC10059913; doi:10.3390/nano13061071)
Supplement: Supplementary file 1 [file nanomaterials-13-01071-s001.zip › nanomaterials-2280205-supplementary.pdf]

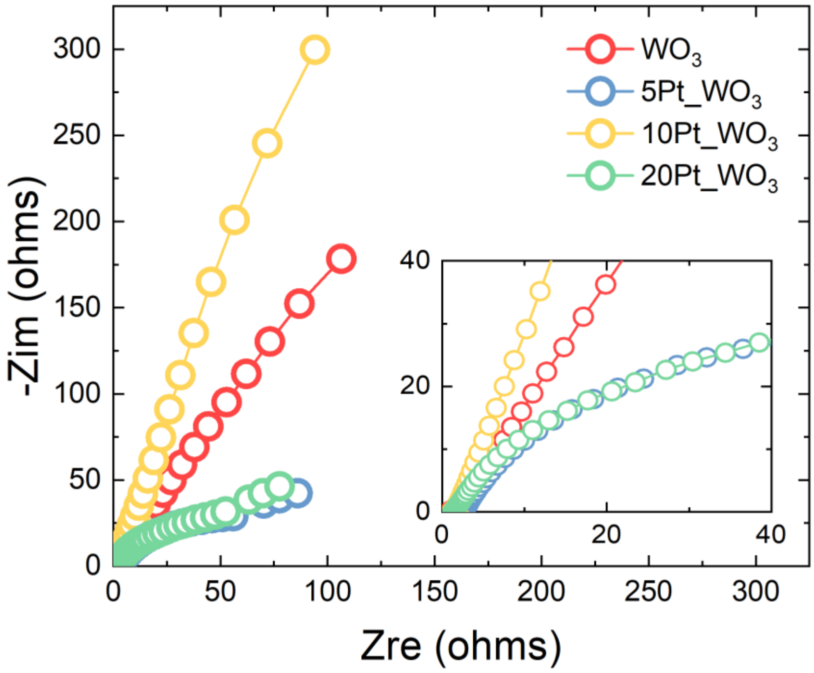

Figure S1: *Nyquist plot obtained from EIS analysis at open circuit potential of the WO_3, 5Pt_WO_3, the 10Pt_WO_3 and the 20Pt_WO_3 electrodes (red, blue, yellow, and green circles, respectively. Inset: magnification of the high frequency region.*

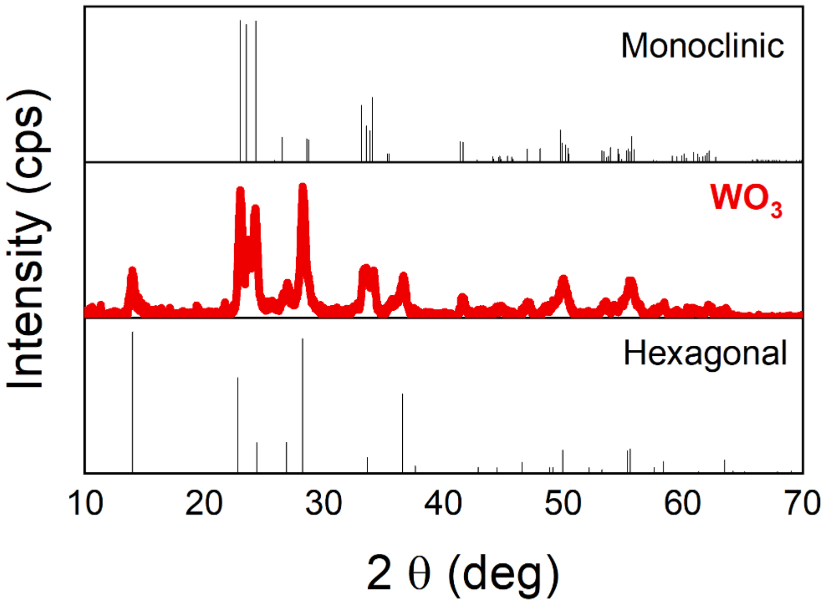

Figure S2: *XRD pattern of the WO_3 nanorods (red line) compared with the characteristic patterns of hexagonal and monoclinic WO_3: Both hexagonal (2Ɵ = 14°, 24.37°, 26.85°, 28.22°, 33.61°, 36.57°, and 49.95°) and monoclinic (2Ɵ= 23°, 23.50°, 24.28°, 33.12°, 33.54°, 33.83°, 34.04°, 49.74°, 55.71°) characteristic peaks appear, thus confirming the formation of stable phase junctions.*

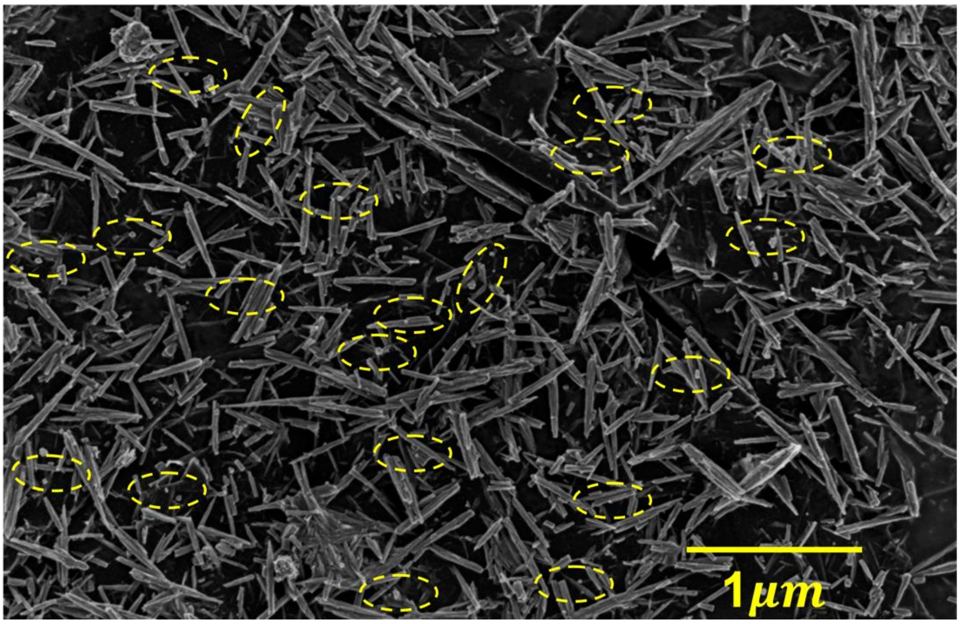

Figure S3: *Low magnification SEM image of the 10Pt_WO_3 electrode in which the most visible Pt nanoparticles are highlighted in yellow.*

RIR methods

The RIR method is used for the calculation of phase composition in a hybrid phase structure, in terms of weight ratio (w%) starting from the intensities of the strongest line of the X-ray diffraction patterns (*I*) as follows [1-2]:

$$W_h = \frac{I_h}{I_h+(I_m/(RIR_m/RIR_h))} \quad \text{(S4)}$$

$$W_m = \frac{I_m}{I_m+(I_h/(RIR_h/RIR_m))} = 1 - W_a \quad \text{(S5)}$$

where *h* and *m* denote the different coexistent hexagonal and monoclinic crystal phases respectively and the *RIR* values depend on the analyzed crystal phases. The RIR values for the *h*- and *m*-WO_3 are 8.33 (PDF #89-4476) and 5.58 (PDF #75-2187). The monoclinic (hexagonal) weight ratio in the WO_3 based nanorods is 56 % (44 %), thus confirming the phase heterogeneity of WO_3 nanorods and the presence of hexagonal and monoclinic crystallites in contact each other to form phase junctions [3].

References

[1] Du, Y.; Hao, Q.; Chen, D.; Chen, T.; Hao, S.; Yang, J.; Ding, H.; Yao, W.; Song, J. Facile Fabrication of Heterostructured Bismuth Titanate Nanocomposites: The Effects of Composition and Band Gap Structure on the Photocatalytic Activity Performance. *Catal. Today* **2017**, *297*, 255–263, doi:10.1016/j.cattod.2016.12.048.

[2] Kang, M.; Liang, J.; Wang, F.; Chen, X.; Lu, Y.; Zhang, J. Structural Design of Hexagonal/Monoclinic WO3 Phase Junction for Photocatalytic Degradation. *Mater. Res. Bull.* **2020**, *121*, 110614, doi:10.1016/j.materresbull.2019.110614.

[3] Mineo, G.; Scuderi, M.; Bruno, E.; Mirabella, S. Engineering Hexagonal/Monoclinic WO3Phase Junctions for Improved Electrochemical Hydrogen Evolution Reaction. *ACS Appl. Energy Mater.* **2022**, *5*, 9702–9710, doi:10.1021/acsaem.2c01383.
